# Investigating the prevalence of hypertension and its associated risk factors in a population-based study: Fasa PERSIAN COHORT data

**DOI:** 10.1186/s12872-020-01797-3

**Published:** 2020-12-01

**Authors:** Mostafa Bijani, Saeed Parvizi, Azizallah Dehghan, Massih Sedigh-Rahimabadi, Mahsa Rostami-chijan, Maryam Kazemi, Mohammad Mehdi Naghizadeh, Alireza Ghaemi, Reza Homayounfar, Mojtaba Farjam

**Affiliations:** 1 Noncommunicable Diseases Research Center, Fasa University of Medical Sciences, Fasa, Iran; 2 Students Research Committee, Fasa University of Medical Sciences, Fasa, Iran; 3 Department of Persian Medicine, Fasa University of Medical Sciences, Fasa, Iran; 4 Healthy Policy Research Center. Institute of Health, Shiraz University of Medical Sciences, Shiraz, Iran; 5 Department of Basic Sciences and Nutrition, Health Sciences Research Center, Addiction Institute, Faculty of Public Health, Mazandaran University of Medical Sciences, Sari, Iran; 6 National Nutrition and Food Technology Research Institute, Faculty of Nutrition Sciences and Food Technology, Shahid Beheshti University of Medical Sciences, Tehran, Iran

**Keywords:** Hypertension, Risk factors, Cohort study

## Abstract

**Background:**

A high prevalence rate of hypertension (HTN) and its subsequent serious complications made this disease a major health-treatment concern in many societies. The current study aimed to investigate the prevalence of hypertension and its associated risk factors in Fasa PERSIAN COHORT in south of Iran in 2019.

**Methods:**

This was an analytical-cross sectional study. The study population were the individuals covered by Fasa cohort. Information of the first phase of Fasa Persian cohort in south of Iran was used in this study. Independent *t* test, chi-square test, analysis of variances, Pearson correlation coefficient, and multiple stepwise regression were used to analyze the obtained data. Thereafter, the data was analyzed using SPSS software version 22, and *P* value < 0.05 was considered as statistically significant.

**Results:**

Out of 10,111 individuals included in the study, 5546 (54.86%) subjects were women and 4565 (45.16%) other were men. The overall prevalence rate of hypertension was estimated as 46.6%. In the present study, (16/1%) of the participants were with HTN stage 1, and (17/9%) of them were with HTN stage 2. The results show that there were significant positive correlations among HTN and age, BMI, HDL, TG, BUN, ALP, smoking, physical activity, cardiovascular diseases, diabetes, and renal failure (*P* < 0.5).

**Conclusions:**

Prevalence of HTN in this study population is considerable. Therefore, it is suggested that health system should program some plans to prevent hypertension’s prevalence and eliminate its risk factors.

## Background

A high prevalence of hypertension (HTN) and its subsequent serious complications made this complication a major problem of health system in many societies [1]. HTN, as a silent assassin, is known as the most prevalent and the most important cause of brain stroke, cardiovascular disease, and heart and renal failures [2]. Moreover, HTN is responsible for 54% and 45% of deaths caused by brain stroke cardiovascular diseases, respectively [3]. Accordingly, the World Health Organization has also introduced HTN as the third leading cause of death worldwide, with one in eight deaths due to high blood pressure [4].

According to the opinion of American society of Hypertension (ASH) and International society of Hypertension (ISH), systolic blood pressure equal to and greater than 140 mmHg and diastolic blood pressure equal to and greater than 90 mmHg can be considered as HTN based on an average value obtained from accurate measurement of blood pressure for at least two times [5]. The results of the study performed by Liew et al. [6] showed that the prevalence of HTN varies based on demographic, cultural, and social factors in different societies, so it is necessary to conduct more comprehensive studies in various regions, especially cohort studies. According to the reports by WHO in 2018, about 40% of individuals aged 25 years old and higher had HTN [7].

In eastern Mediterranean countries and Middle East, the prevalence of HTN in various assessments was reported to be between 17 and 39%, and rapid social and economic changes of these countries in recent decades resulted in a high prevalence of the HTN [8]. The results of a study conducted in Iran reported the prevalence of HTN as 39.4% in age group of 15–65 years old [9]. According to the results of a cohort study, the prevalence of HTN was reported as 41.8% in Golestan province [10]. In another study, the prevalence rates of HTN in urban areas of Golestan and in Gorgan city were reported as 32% and 25%, respectively [11].

Since HTN is considerably prevalent in most communities, and its prevalence in various regions varies due to demographic, cultural, and social factors. Since there is no study performed in Iran based on the results obtained from cohort data to date, and besides, due to the reason that the identification of risk factors of HTN will play important role in programming for better prevention, control, and treatment of this complication, this study aimed to investigate the prevalence of hypertension and its associated risk factors in Fasa PERSIAN COHORT in south of Iran in 2019.

## Methods

The current study was an analytical-cross-sectional study. The study population were the individuals covered by Fasa Cohort in south of Iran [12].

**Fig. 1:**
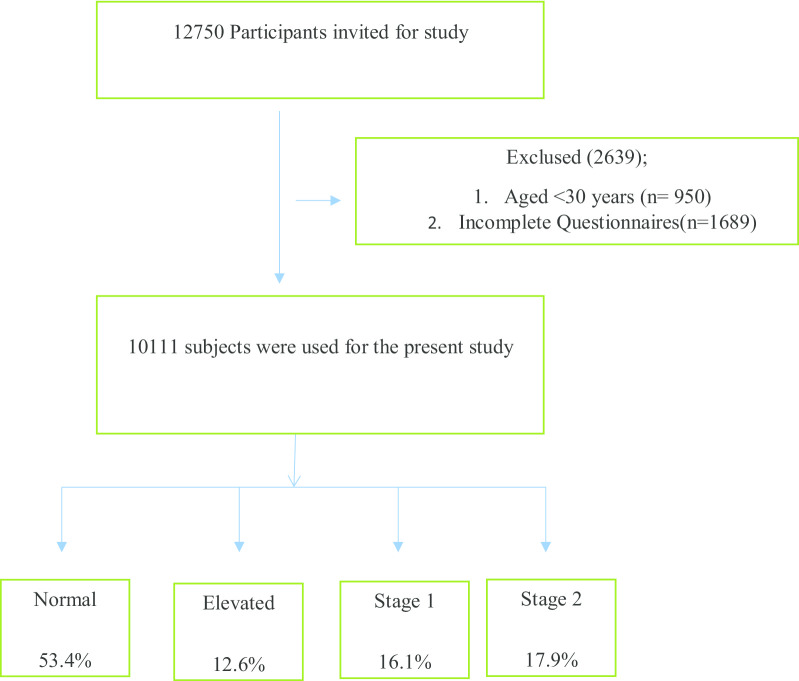
Participant recruitment flow diagram

The data used in this research were driven from the base-line survey of the PERSIAN (Prospective Epidemiological Research Study in Iran) cohort Study (Fasa noncommunicable disease cohort study) [12]. Moreover, the subjects were selected according to consensus sampling. Accordingly, a total number of 10,111 participants aged 30 years old or above were recognized as eligible and then invited to participate in this study (Fig. 1). In order to gather data, a questionnaire consisting of demographic data and risk factors of HTN, was used.

In addition, anthropometric indices (measurement of height-weight and waist circumference) as well as blood pressure measurement were used. In order to measure blood pressure, the average of two times measurement of blood pressure was considered as the criterion. Thereafter, in terms of the 2017 ACC/AHA guideline, the participants were divided into four groups as follows: those with systolic blood pressure less than 120 mmHg and diastolic blood pressure less than 80 mmHg were considered as normal blood pressure, those with systolic blood pressure of 120–129 mmHg and diastolic blood pressure less than 80 mmHg were considered as the elevated hypertension, the subjects with systolic blood pressure of 130–139 mmHg and diastolic blood pressure of 80–89 mmHg were considered as stage 1 hypertension, and finally those with systolic blood pressure equal to and greater than 140 mmHg and diastolic blood pressure equal to and greater than 90 mmHg were considered as stage 2 hypertension [13].

Independent *t* test, chi-square test, analysis of variances, Pearson correlation coefficient, and multiple stepwise regression were used to analyze the obtained data. Notably, the data were analyzed using SPSS software version 22, and *P* < 0.05 was considered as statistically significant.

### Ethical considerations

All the participants gave written informed consent to participate in the study. The present study was conducted in terms of the principles of the revised Declaration of Helsinki, which is a statement of ethical principles that directs physicians and other participants in medical research involving human subjects. The participants were assured about their anonymity and confidentiality of their information. Moreover, the study was approved by the Institutional Research Ethics Committee of Fasa University of Medical Sciences, Fasa, Iran (code: IR.FUMS.REC.1398.063).

## Results

Out of 10,111 participants, 5546 (54.86%) subjects were women and 4565 (45.14%) others were men. The average age of the participants was 34.48 ± 8.4 years old. Furthermore, the mean age of male subjects was 49.58 ± 9.4 years old. Accordingly, the youngest man was 35 years old and the oldest one was 87 years old. In female individuals, the mean age was 49.64 ± 9.58 years old, the youngest woman was 37 years old, and the oldest one was 79 years old. The mean of systolic blood pressure in male participants was 110.52 ± 17.56 and diastolic blood pressure was 74.36 ± 11.84. The mean of systolic blood pressure in female subjects was 136.00 ± 19.69 and diastolic blood pressure was 83.01 ± 11.52. The total means of systolic and diastolic blood pressure were 111.36 ± 18.51 and 74.65 ± 11.99, respectively (Table 1).

**Table 1:** Mean systolic and diastolic blood pressure (mm hg) by age and sex

Age groups (years)		Systolic BP (mean ± SD)			Diastolic BP (mean ± SD)		
Age	N	Male	Female	Total	Female	Male	Total
35–44	4020	105.24 ± 13.25	104.89 ± 14.83	105.05 ± 14.13	72.12 ± 11.36	72.47 ± 10.82	72.28 ± 11.11
45–54	3154	109.20 ± 16.53	112.75 ± 18.41	111.20 ± 17.70	75.68 ± 12.18	74.11 ± 11.96	74.99 ± 12.11
55–64	2329	118.08 ± 20.17	119.97 ± 21.05	119.08 ± 20.66	77.58 ± 12.33	76.97 ± 12.63	77.29 ± 12.47
65–74	569	122.37 ± 19.81	124.47 ± 22.04	123.65 ± 21.20	78.34 ± 11.92	78.04 ± 11.76	78.22 ± 11.85
Over 75	39	133.07 ± 22.49	136.00 ± 19.69	133.82 ± 21.59	83.01 ± 11.52	79.48 ± 13.45	80.38 ± 12.94
Total	10,111	110.52 ± 17.56	112.05 ± 19.22	111.36 ± 18.51	74.88 ± 12.11	74.36 ± 11.84	74.65 ± 11.99
*P* value		F = 148.60	F = 191.66	F = 334.31	F = 55.81	F = 32.78	F = 85.75
		df = 4	df = 4	df = 4	df = 4	df = 4	df = 4
		*P* ≤ 0.001	*P* ≤ 0.001	*P* ≤ 0.001	*P* ≤ 0.001	*P* ≤ 0.001	*P* ≤ 0.001

The overall prevalence rate of hypertension was calculated as 46.6%. In the present study, (16/1%) of the included subjects were with HTN stage 1, and (17/9%) of them were with HTN stage 2 (Tables 2, 3). Tables 4 and 5 show the associated factors of hypertension. In this regard, it was indicated that Age, BMI, Hip circumference, HDL, TG, BUN, ALP (Alkaline phosphatase), Smoking, Physical activity Diabetes, Cardiac ischemic, Renal Failure, and Brest feeding were significantly associated with the hypertension status of the studied subjects (*P* < 0.05) (Tables 4, 5).

**Table 2:** Prevalence systolic of hypertension by sex and age groups among the study subjects

Age groups (years)		Male				Female			
Age	N	Normal	Elevated	Stage 1 HTN	Stage 2 HTN	Normal	Elevated	Stage 1 HTN	Stage 2 HTN
35–44	4020	1669 (90.6)	84 (4.6)	54 (2.9)	35 (1.9)	1924 (88.4)	94 (4.3)	91 (4.2)	68 (3.1)
45–54	3154	1125 (81.7)	67 (4.9)	99 (7.2)	86 (6.2)	1303 (73.3)	128 (7.2)	163 (9.2)	183 (10.3)
55–64	2329	678 (62)	87 (8)	154 (14.1)	174 (15.9)	728 (58.9)	120 (9.7)	161 (13)	227 (18.4)
65–74	569	112 (50.5)	30 (13.5)	36 (16.2)	44 (19.8)	178 (51.3)	29 (8.4)	54 (15.6)	86 (24.8)
Over 75	39	10 (34.5)	3 (10.3)	6 (20.7)	10 (34.5)	2 (20)	0 (0)	3 (30)	5 (50)
Total	10,111	3594 (78.8)	271 (5.9)	349 (7.6)	349 (7.6)	4135 (74.5)	371 (6.7)	472 (8.5)	569 (10.3)
*P* value	–	χ^2^ = 533.64				χ^2^ = 542.16			
		df = 12				df = 12			
		*P* ≤ 0.001				*P* ≤ 0.001

**Table 3:** Prevalence diastolic of hypertension by sex and age groups among the study subjects

Age groups (years)		Male			Female		
Age	N	Normal/elevated	Stage 1 HTN	Stage 2 HTN	Normal/elevated	Stage 1 HTN	Stage 2 HTN
35–44	4020	1212 (65.8)	469 (25.4)	162 (8.8)	1432 (65.7)	556 (25.5)	190 (8.7)
45–54	3154	845 (61.4)	343 (24.9)	189 (13.7)	961 (54.1)	538 (30.3)	278 (15.6)
55–64	2329	542 (49.5)	343 (31.4)	209 (19.1)	618 (50)	374 (30.3)	243 (19.7)
65–74	569	100 (45)	77 (34.7)	45 (20.3)	158 (45.5)	120 (34.6)	69 (19.9)
Over 75	39	13 (44.8)	9 (31)	7 (24.1)	3 (30)	3 (30)	4 (40)
Total	10,111	2712 (59.4)	1241 (27.2)	612 (13.4)	3172 (57.2)	1591 (28.7)	784 (14.1)
*P* value	–	χ^2^ = 137.86			χ^2^ = 122.68		
		df = 12			df = 12		
		*P* ≤ 0.001			*P* ≤ 0.001

**Table 4:** The relationship between variables affecting systolic blood pressure using stepwise regression

Variable	Unstandardized Coefficients		Standardized Coefficients	t	Sig
	B	Std. Error	Beta		
(Constant)	45.302	1.694	−	26.740	< 0.001
Age	.668	.021	.345	31.956	< 0.001
BMI	.816	.037	.213	22.132	< 0.001
Hip circumference	.136	.043	.065	3.138	.002
HDL	.150	.011	.129	13.880	< 0.001
TG	.021	.002	.095	10.071	< 0.001
ALP (Alkaline phosphatase)	.017	.002	.064	6.843	< 0.001
Active smoker	− 1.517	.402	− .036	− 3.772	< 0.001
Menopause	− 1.878	.495	− .042	− 3.795	< 0.001
Cardiovascular diseases	1.493	.549	.025	2.718	.007
Renal failure	− 3.963	1.683	− .021	− 2.355	.019
Diabetes	1.116	.518	.020	2.152	.031
Calcium	.001	.000	.020	2.251	.024
Magnesium	.002	.003	.035	.787	.031
Potassium	.000	.000	− .063	− 1.369	.017
Sodium	.000	.000	.029	3.010	.003
Physical activity	− .033	.015	− .020	− 2.225	.026
Brest feeding	− 1.783	.327	− .028	− .2.98	0.036

Dependent variable: SBP

**Table 5:** The relationship between variables affecting Diastolic blood pressure using stepwise regression

Variable	Unstandardized Coefficients		Standardized Coefficients	t	Sig
	B	Std. Error	Beta		
(Constant)	39.051	1.042	–	37.479	< 0.001
BMI	.568	.025	.228	23.011	< 0.001
Age	.234	.014	.187	17.031	< 0.001
Hip circumference	.083	.029	.061	2.811	.005
HDL	.100	.007	.133	13.646	< 0.001
TG	.015	.001	.101	10.306	< 0.001
ALP (Alkaline phosphatase)	.011	.002	.065	6.609	< 0.001
Calcium	.001	.000	.041	4.320	< 0.001
Magnesium	.002	.003	.035	.787	.043
Potassium	.000	.000	− .063	− 1.369	.013
Active smoker	− 1.289	.279	− .046	− 4.616	< 0.001
Renal failure	− 2.233	1.133	− .018	− 1.972	.049
Menopause	− 1.368	.330	− .047	− 4.150	< 0.001
Brest feeding	− .028	.003	− .026	− 2.843	.024

Dependent variable: DBP

## Discussion

Hypertension is the most important and prevalent chronic disorder, which still remains a major health concern despite many medical progresses. Given the prevalence of this disease and its serious subsequent complications, the current study aimed to investigate the known risk factors for hypertension as well as studying their associations in study population of the Cohort in south of Iran in 2019.

Number of adults with hypertension in Iran reached 9 million and 7 hundred thousand individuals in 2015, out of whom, more than 5 million and 2 hundred thousand were man and more than 4 million and 5 hundred thousands were woman. Number of individuals with HTN in Iran has also increased from 4 million individuals (2 million and 1 hundred thousand men and one million and 9 hundred thousand women) to more than 9.7 million in 2015. Correspondingly, the majority of this increase was resulted from an increase in population of Iran and increase in life expectancy of the Iranian population [14].

The prevalence of HTN varies in various studies, which in part could be attributed to differences in time, region, and the population’s ethnics of the studies. However, these different numbers can also be resulted from a mistake or bias. As an example, in the sugar and lipid study of Tehran, the prevalence of HTN in old ages was reported to be about 47% [15], and in the study by Sharifi, it was reported as 60% [16].

However, there are some studies that estimated this rate as 25% [17]. In addition, this value in the current study was obtained as 46/6% based on the 2017 ACC/AHA guideline. The results of the cohort studies, similar to our study, commonly respond better and more accurate for prevalence studies, since they were not affected by bias.

Regarding the effective factors on blood pressure, the most significant association was observed with blood pressure in univariate associations, BMI, BUN, diabetes, cigarette use, and breastfeeding duration.

The study performed by Rao et al. [18] in India showed that prevalence of the elevated blood pressure in Bambaei was about 43%, and the most important factors associated the HTN in their study were age over 40 years old, male sex, having diabetes, BMI greater than 25, and a family history of HTN.

The study by Fawokan et al. [19], which investigated the effective factors on blood pressure in pediatrics as a systematic review, introduced some factors such as sex, being overweight, economic and social status, educational level of parents, being stable, using tobacco and alcohol, and finally dietary regimen among the factors effective on the increased susceptibility to HTN.

The study by Anteneh et al. [20] have also investigated the effective factors on HTN in Ethiopia. In this study, its prevalence rate was reported as 25.1%, and age, tobacco use, physical activity, diabetes history, salt intake, and BMI were introduced as the most important factors related to HTN.

The study by Patra and Bhise also investigated the factors related to HTN in India population and then reported general prevalence of HTN about 25%. Moreover, this study reported some factors associated with the disease as age, sex, marital status, habitat, socioeconomic status, unhealthy behaviors such as tobacco and alcohol, and a high BMI [21]. The results of our study propose more variables compared to most of the previous studies done in the environment similar to the region of our study. The results obtained by Cicero et al. in Italy and the study of Bawazier et al. in Malaysia showed that there was a direct and significant relationship among smoking, lack of physical activity, high BMI, high LDL and low HDL levels, and high triglyceride levels with blood pressure, which is consistent with the results of the present study. Additionally, a significant relationship was observed between uric acid level and blood pressure in the two above-mentioned studies. Accordingly, this variable has not been studied in the present study [22, 23].

The results obtained from the study by Saju et al. in India and the study by Gao et al. in China showed a direct and significant relationship among smoking, lack of physical activity, diabetes, and ischemic heart disease and blood pressure. In addition, in the present study, the above-mentioned factors had significant relationships with blood pressure [24, 25]. Rabi stated that since most blood pressure factors are modifiable, lifestyle and diet modifications can be considered as the most important and effective measures for the prevention and subsequent reduction of nasal side effects of hypertension. Therefore, it is necessary for health system managers to use the necessary programs, in order to correct and eliminate the risk factors for high blood pressure and it also is important to evaluate the effectiveness of executive programs continuously [26].

Numerous biochemical and nutritional indices were entered into the study, and for the first time, by comparing similar studies, all the medications related to HTN that might affect blood pressure status were investigated (potency of decrease and increase of blood pressure as a side effect). Afterward, they were entered in the multi-variate analysis to attenuate their effects on blood pressure. Regarding the analysis of blood pressure, in order to achieve more accurate results and to control the effects of high and low pressures on the results in some groups, regression analysis was used, and as the results show, some of the findings are fully consistent with findings of similarly performed studies. For example, BMI and/or waist circumference, which are known as indices of obesity, have almost played critical roles in most sex and blood pressure groups, and were considered as significant risk factors for blood pressure.

Of course, it should not be neglected that waist circumference in similar previous studies was not used, and index of abdominal obesity that is represented by waist circumference, was neglected in other studies. While in our study, for SBP and DBP in women, after the modification of many known variables, waist circumference showed a strong association with HTN. As stated earlier in the other studies, diabetes is considered as a risk factor for HTN, and in this regard, blood sugar showed a strong association with HTN in our study.

Alkaline phosphatase (ALP) is considered as an index of hepatic function that showed a considerable association with HTN specially SBP, which was also pointed in few studies. Regarding the variables related to dietary regimen, as expected, sodium is associated with HTN, and almost all the previous studies had consensus on it; however, an interesting point in our study was its weak association compared to other variables.

While opposite to our expectation, calcium and potassium were pointed as anti-HTN factors in many studies and were considered as the components of DASH diet (Dietary Approaches to Stop Hypertension) [27, 28], they were indicated to be blood pressure elevators in our study. However, there is a need for more investigating this finding to better clarify the underlying reason.

Another component of DASH diet that is considered as a decreasing factor of HTN, is magnesium, which in our study was shown as a preventive factor, and ths is fully consistent with the findings of other studies [29, 30].

Another considerable point in our study was breastfeeding duration in women, which is a risk factor for susceptibility to HTN, approximately opposite to the other studies that introduced breastfeeding as a preventive factor for HTN [31]. Accordingly, our study showed that breastfeeding duration can increase the risk of HTN.

Although the study by Struebe et al. [32] pointed to this tip, they explained the underlying cause so that in 12% of mothers who reported this increase in blood pressure, breastfeeding duration was lesser than the expected time, and they recommended that thorough breastfeeding may prevent this elevation in blood pressure.

This is the point that should be considered in further studies; however, average breastfeeding duration in our study was considerable as 86.4 ± 58.8 months compared to other studies. In this regard, the reason for this considerable and different finding might be due to the long duration of breastfeeding, which we know that it can consequently cause considerable health risks to mother due to mother’s reservoir loss.

### Limitations and strengths of the study

Strength of this study was that it is the first community-based cohort study performed in South Iran. One limitation of the study was that the 2017 ACC/AHA Guideline was used in the present study to stage blood pressure; therefore, it is recommended to use other guidelines in future studies and also to perform studies in other countries.

## Conclusions

The findings of the current study showed a high prevalence of HTN in the study population; therefore, it is necessary to perform the required programs by the managers and policy makers of the health treatment system with the aim of screening and identifying hazardous factors, promoting self-care behaviors and management, and controlling HTN. In addition, since HTN is affected by cultural, social, and economic factors, it is necessary to conduct some studies in other countries, especially in cohort format, to investigate the prevalence as well as hazardous factors of HTN.

## Data Availability

The datasets used and/or analyzed during the current study are available from the corresponding author on reasonable request.

## Abbreviations

HTN: Hypertension
SBD: Systolic blood pressure
DBP: Diastolic blood pressure
